# Research on E-Commerce Database Marketing Based on Machine Learning Algorithm

**DOI:** 10.1155/2022/7973446

**Published:** 2022-06-29

**Authors:** Nie Chen

**Affiliations:** Department of Electronic Commerce, Zhejiang Business Technology Institute, Ningbo 315012, China

## Abstract

From simple commercial relations to complex online transactions at this stage, it not only highlights the progress of science and technology, but also indirectly explains the diversified evolution of marketing methods and means. In marketing, database marketing has been favored by more marketers with its low cost and high efficiency and has become the “rookie” in marketing in recent years. However, as a kind of prediction and ferry, database marketing tends to be applied after simple data analysis in unpredictable market and in practice. In contrast, database marketing combined with machine learning algorithms has always been a depression in the marketing field. Therefore, this paper takes e-commerce as the research object and carries out database marketing research based on machine learning algorithm from four stages: theoretical preparation, status analysis, model construction, and results application. Firstly, the connotation, advantages, and specific operation procedures of database marketing are discussed. At the same time, four excellent machine learning algorithms including logistic regression, random forest, support vector machine, and gradient boosted decision tree (GBDT) are selected to explain the basic principles and algorithm introduction, respectively, laying a theoretical foundation for the model training chapter. Secondly, it analyzes the current situation of e-commerce from the distribution of marketing objects, the proportion of marketing channels, and the composition of marketing methods and finds new marketing ideas based on the main problems existing at the present stage of database marketing using machine learning algorithm. Thirdly, on the premise of marketing ideas, data acquisition, data processing, and positive and negative sample setting. At the same time, four machine learning algorithms are used to combine features from the perspectives of consumers, stores, and the relationship between consumers and stores. Finally, by substituting the predicted sample into the model for testing, the crowd whose predicted score is between 80 and 99 is selected to be put into the market as the model predicted crowd, and it is proposed that e-commerce should mainly adopt the database marketing method of model prediction. On the one hand, machine learning algorithm can solve the problem of uneven distribution of marketing objects, and on the other hand, it can effectively prevent the loss of potential consumers. In addition, the application strategy of optimizing other database marketing methods and assisting model prediction to improve marketing effect is also put forward.

## 1. Introduction

In the 21st century, under the new trend driven by big data, database marketing is favored by e-commerce and new retail industry. Because rich consumer data provide the data foundation for database marketing, statistical analysis, machine learning algorithm, and data mining technology based on this development provide the technical foundation for customer insight for electric stores, which can be applied in almost every link of marketing activities. However, at present, due to information overload, consumers visit multiple platforms and various fields. Although consumers focus their time and energy on discovering and reaching many commodities, they fail to include all of them [1]. At the same time, businesses should also look for more effective strategies to accurately target consumers in the face of increasing commercial competition. The development of enterprises and the profits of stores are inseparable from the accurate positioning of people. However, the complexity of the number and variety of commodities has brought challenges to this refined database marketing. Therefore, “database marketing” has become a major topic and practical problem that Chinese and foreign academic circles and large enterprises continue to pay attention to. Consumer analytics have been at the center of the big data revolution, and technology has evolved to help capture data on consumer behavior in real time. Developed countries are undoubtedly still pioneers in this area, applying machine learning algorithms and data mining techniques to marketing and other fields. Its theoretical guidance and practical experience in database marketing have very important reference value for the development of Chinese enterprises and e-commerce platforms [2]. Through combing the literature at home and abroad, it can be concluded that the key of database marketing lies in the positioning of target consumers. With the advent of the era of big data and the popularization of computer technology, the process of database marketing defining consumer groups is regarded as a supervised learning problem in the existing literature [3]. That is, according to the collected data, groups are classified in combination with the differences in certain consumption characteristics, and then, it is predicted whether this category will buy goods and the probability of buying goods. A large number of foreign literature have applied support vector machines, decision trees, and other machine learning algorithms, but there is still a gap between China and foreign developed markets. It is mainly reflected in the following aspects: firstly, the domestic market situation and the consumption concept in inland areas limit the function of database marketing. Secondly, it is the lack of high practicality and high operational macro database marketing strategy. Thirdly, there is a lack of machine learning algorithms and data analysis and data mining technical professionals, resulting in the lack of corresponding technical support for database marketing. The marketing strategy only stays at the level of intuitive and simple data analysis, and has not correspondingly improved the marketing level of the enterprise, rather than the marketing cost, because the positioning accuracy of target customers has become higher.

With the advent of the data age, database marketing has changed to a certain extent. Great changes have taken place both in the form of data presentation and in the statistical methods adopted by database marketing. We can no longer simply understand the database marketing as the traditional database marketing without data interaction, but it is defined as the whole database marketing mode with various functions based on the development level of information technology [4]. On the one hand, the data form in the context of big data has the characteristics of a large amount of data, complex data structure, and diversified data modes. The main reasons are as follows: first, the amount of information is increasing rapidly with the popularization of the Internet and the development of e-commerce; second, visible and unknown market data and information can be turned into public information through multimedia; third, consumers' consumption ideas and preferences are displayed in different ways on other websites or platforms. Not all of the data are useful, and “information overload” needs to be identified, which makes the acquisition and storage of data more challenging. On the other hand, in the context of big data, data marketing methods tend to be more predictive algorithms. Traditional database marketing uses certain descriptive statistics methods to describe from different perspectives, among which the most commonly used is sales channel analysis, so as to decide whether to invest corresponding costs to plunder other channel resources according to the comparative analysis results. The data involved are only the behavior data in the customer information of the database, and the data are small and easy to operate. However, in the context of big data, the platform will generate a large amount of data every day. If this simple method is still used for analysis and operation, “flooding” will occur, with a large amount of data, high cost, and dilution of the effect of delivery. However, the current method of processing big data is computer cluster and compatible technology. Efficient intelligent grid computing, cloud computing, and machine learning prediction algorithms have emerged to extract effective information and knowledge from massive data in a timely and accurate manner [5–7].

The combination of machine learning algorithm and database marketing arises because of the complexity of data forms, the diversity of data structure, and the limitation of data marketing methods. First, machine learning algorithms need to be applied to database marketing. On the one hand, the manifestation of marketing database data is diversified. Traditional statistical methods can only analyze numerical data, and it is difficult to process data of text, audio, and video, thus restricting the development of database marketing. On the other hand, as a data analysis method, machine learning algorithm has its “uniqueness.” By organizing information in batches and purposefully, machine learning can uncover potential information and provide it to users or derive more valuable information. These features provide a convenient channel for database marketing, which can not only be applied to acquire new consumers and establish long-term relationship with consumers but also enhance consumer value. Therefore, database marketing in conformity with the trend of modern e-commerce times to achieve more efficient marketing basis must be supported by machine learning algorithms. Second, the integration of machine learning is needed to improve database marketing. In specific practice, there are only two purposes of e-commerce database marketing, which are to recruit new customers by means of contact conversion, cross-platform backflow, cross-category plunder, and competitive product analysis [8]. By maintaining customer relationship, calculating repurchase period and counting repurchase rules, the purpose of operating regular customers is achieved. In view of different goals, different means are adopted to achieve them. However, with the arrival of big data and the wide application of machine learning algorithms, traditional database marketing methods cannot fully meet the development of Tmall stores. In order to have certain competitive advantages in the market and play a greater role in the Chinese market, e-commerce must carry out targeted database marketing by integrating big data and upgrading marketing methods and ideas with machine learning algorithm so as to increase source and reduce expenditure, reduce costs, improve marketing effects, and improve database marketing methods [9].

Therefore, this paper takes e-commerce as the research object, effectively uses e-commerce information to maintain and enhance the emotional bond between stores and consumers, while avoiding open confrontation with competitors, and explores more refined database marketing application strategies of e-commerce [10]. Compared with traditional database marketing, database marketing based on machine learning algorithm can more easily establish a stable relationship with customers, reduce the cognitive differences between its customers, and provide more accurate decision-making direction and development strategy. On the one hand, constructing prediction model based on machine learning algorithm can achieve higher precision of target customer positioning and improve the effect of database marketing. On the other hand, it provides effective decision support for marketing managers and has a high degree of interpretability. The innovation of this paper lies in the deepening of database marketing research from the practical application level. At present, the domestic researches on the combination of machine learning algorithm and database marketing are mostly from the perspective of macro analysis and microdescription but lack case analysis. This paper puts forward database marketing strategies for specific online stores, close to the development of database marketing, and promotes the deepening of database marketing research from the practical level.

## 2. Overview of Database Marketing

### 2.1. Connotation and Advantages of Database Marketing

**Figure 1:**
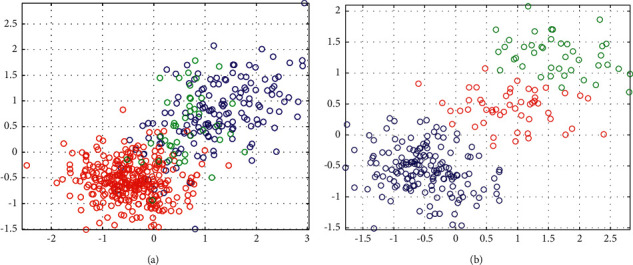
Data clustering diagram. (a) Before cluster analysis and (b) after cluster analysis based on t-SNE.

Database marketing is a marketing method by collecting and accumulating a large amount of consumer information, predicting how likely consumers are to buy a certain product after processing, and using this information to accurately position the product, and making targeted marketing information so as to persuade consumers to buy products [11]. Database marketing is the process of establishing, maintaining, analyzing, and applying customer data and related information in order to establish contact with customers, promote transactions, and maintain customer relations. The data clustering diagram is shown in Figure 1.

With the gradual rise and maturity of the Internet, IT, and database technology, database marketing, as a marketing model and one of the most powerful marketing tools for enterprises to pursue profit maximization, can more accurately locate consumers' consumption psychology and habits so that enterprises can realize how to better plan through the rational use of database marketing, in order to meet the needs of consumers, to provide consumers with better, more perfect service [12]. Database marketing is relationship marketing in the field of industry marketing and direct marketing development, which is based on modern information technology, marketing, and statistics and forms a borderline science, as a kind of brand-new way of enterprise management and database marketing in the technical support fixation method, in a marketing tool, and provides a broad marketing platform. As a dynamic data management system, database marketing has two main functions [13]. First, database marketing can reasonably focus on the analysis of consumers of different qualities, such as analyzing the shopping data of old customers to get the consumption direction and preference, so as to achieve the purpose of establishing long-term good relations with them. Second, database marketing through the establishment of specific enterprises and specific groups of the database can be achieved: (1) timely interaction and communication with consumers, (2) making the brand more competitive in the market, (3) increasing turnover, and (4) making the sales process faster and simpler. Database marketing has been successful in practice and operation because of its desirability and superiority. The advantages of database marketing are mainly reflected in the following aspects.

Firstly, database marketing can effectively collect, store, and use data for individuals, which can realize the effective combination of the whole and the individual. Database marketing can not only carry out customized marketing for individuals with characteristics but also integrate the purposes and ways of individual consumption in a period of time [14]. In this way, enterprises can quickly get in touch with consumers and have a deeper understanding of consumer psychology. The seemingly complex “individual” marketing model and data statistics can be easily reflected through database marketing. The advantage of this mode is that it can clearly and reasonably aim and locate the target, and the results obtained are traceable, easy to query, and have huge development and competitive advantages.

Secondly, database marketing deepens the marketing idea of “customer is God.” Marketers should take consumer value as the core without harming their own interests. Although both technology and services were backward in the past, consumers could also enjoy the products and services provided by marketers, but the difference was that all consumers were treated as ordinary individuals without any difference. However, with the rapid development of information technology, in order to better serve consumers, consumers with different characteristics and different buying habits are usually targeted for marketing. In this way, for marketers, costs are saved, while consumers also get better service.

Thirdly, database marketing can provide “a lot of customization” to consumer groups. Database marketing based on modern information technology has been able to provide “private customization” for individual consumers, and it can also carry out batch customization for all individuals, which makes “one-to-one service” more generalized in practice. Through the consumer shopping data, consumer behavior data, and consumer characteristic data collected in the existing database, marketers classify individuals according to different labels and provide differentiated products and services for each category so that consumers can find target products according to their own needs.

Fourthly, database marketing can provide guidance to channel members and improve channel operation efficiency. In modern marketing mode, in order to establish a perfect supply and marketing chain, manufacturers, retailers, and consumers must be linked. Retailers provide sales data to manufacturers, who organize them into databases and purchase raw materials and produce goods in a planned way according to analysis and calculation. Retailers can obtain consumers' consumption data through various channels and establish lasting relationships with consumers by providing satisfactory services so that consumers can develop into “regular customers” and “repeat customers” and avoid turning to other competitors. Manufacturers can also obtain data to provide sales guidance to distributors or retailers, which can effectively avoid power bias in the channel and benefit all aspects of the channel.

### 2.2. Basic Procedures of Database Marketing

**Figure 2:**
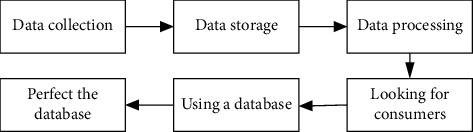
Database marketing basic operating procedures.

Database marketing can not only monitor quality comprehensively and manage it but also develop into a brand new marketing mode through the media of information technology and reach a new height in the process of continuous practice and inspection [15]. Database marketing is becoming more and more perfect so that it is widely used, such as in commercial services, customized services, marketing under big data, public welfare undertakings, and even in industrial production and fund-raising and other fields, and database marketing can be seen everywhere. Since database marketing can replace the traditional marketing, it is necessary to rational macro monitoring and careful market planning. In order to eliminate the uncertain influence and factors of the market, database marketing can abstract quantification of various problems in the market for more accurate classification and positioning in order to develop efficient marketing programs. In addition, database marketing needs to establish “trust” and “intimacy” with consumers, which requires marketers to clarify the relationship between human and machine and also data and market from the perspective of humans so as to establish a unique and personalized marketing system. Take “people” as the core, build a bridge between consumers and marketers, weigh the rights of consumers and marketers, and make better use of the database for marketing [16]. The database marketing basic operating procedures are shown in Figure 2, and the basic procedures for database marketing can often be summarized as follows.

Firstly, data acquisition. It mainly collects data from three aspects: consumer information, product information, and competitor information. The marketing database mainly includes consumer information, including basic information of consumers, consumers' preferences and behavior patterns, business interaction between flagship stores and consumers, and consumers' past purchase behaviors, which are generally obtained through customers' own registration information and behavior information recorded in the background. In the product information, the basic information of the product, supply, marketing, and inventory is generally provided by the corresponding department of the flagship store. The feedback mechanism provided by the platform will collect the product and service situation and consumer opinions [17]. The information about competitors generally includes who the competitors are, their business scale, product composition, the number and amount of people flowing into and out of the brand, the promotion mechanism and membership mechanism of competitors, and the reaction of consumers, which are collected through the third-party industry data release, third-party statistical platform detection, competitive user customer base survey, and other methods. In the process of data collection, it is necessary to avoid information clutter and disorder and ensure the timeliness, accuracy, authenticity, and availability of the collected information.

Secondly, data storage. After obtaining data for different purposes through different channels, it must be saved in a safe and effective way, and the mode of saving is generally selected as database storage mode. First of all, the customer information collected by the database service company is accurately stored in the specially established database, and then through the data fusion channel, the product information data and competitor information data are input into the database for storage in accordance with the standard format. Database can be used to analyze data information and make certain demand prediction on this basis so as to produce more decision information.

Thirdly, data processing. Data processing technology is used to synthesize disordered, nonstandard, and different attributes of the original data into organized data; timely grasp consumer demand changes; and according to the trend of change, timely adjust the business direction, capture marketing opportunities, but also to meet the other needs of each business department. Because the original data stored by various enterprises are not only used for marketing, it applies the information obtained from database analysis to product research and development, product improvement, customer relationship management, and other fields. Therefore, for the data after data processing, centralized control should be achieved while ensuring independence, data consistency, and universality of application.

Fourthly, find consumers. In a specific period of time, consumers' buying behavior often has some common characteristics, such as interest, income level, and preference; the marketing personnel will label the buyer's information, form the customer's purchase portrait, grasp the buyer's own characteristics, attributes, and purchase intention, and find the target group and target the ideal customer through marketing means on the basis of the destination.

Fifthly, use a database. Data can be used in many ways according to the specific business: judging consumers' consumption level and consumption ability according to consumption records; according to the characteristics of consumers, to determine the production of advertising materials and advertising; in addition, by determining the store membership mechanism and deciding which part of people to be included as members, the turnover rate of members will be improved to determine the store promotion mechanism, determine which promotion form is more popular, and achieve the store sales target during the promotion while maximizing profits. In this paper, e-commerce data are mainly used to build feature vectors and models to predict target buyers more accurately.

Sixthly, improve the database. The improvement of database mainly starts from three aspects: first, with the increase of customers in daily operation, the database is constantly enriched and improved; second, collect valid information related to lucky draw sales activities, coupon feedback, product reviews, and so on and further supplement the contents of the database; third, cross-platform advertising crowd return, integration of external data, and make the database more diverse and complete. In short, with the continuous accumulation of daily transactions, multiplatform, and multifield data fusion, the amount of collected information is increasing, the data structure is more diversified, and the database is also improved. A more complete database can provide a more accurate analysis basis, which is conducive to more accurate targeting of target consumers.

Database marketing does not have enough understanding of the potential market, the development prospect, and the use of marketing methods. This has led enterprises to be in the discussion and exploration stage for a long time. During this period, the direct value of database marketing has only produced it, but its potential value has been ignored [18].

**Figure 3:**
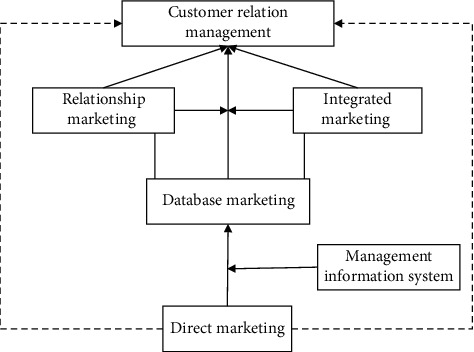
Database marketing and related conceptual models.

This paper mainly builds a model based on machine learning algorithm to predict consumers' purchasing behavior on Taobao in a certain period of time [19]. For this purpose, the data of brand data bank in the past year are selected for analysis, which mainly includes consumer portrait information, historical transaction data, consumer browsing log, and product data. The database marketing and related conceptual models are shown in Figure 3.

*Consumer portrait information*. Consumer portrait information refers to the specific characteristics of consumers; usually, the consumer portrait information collected by the database is divided into two categories [20]. There are 15 basic labels, including gender, age, purchasing power, average monthly spending, geographic location, and type of phone. The other category is called industry label, which mainly includes annual consumption amount, consumption frequency, and consumption preference of the industry. Through the collection of consumer portrait information, the characteristics of potential buyers can be deeply explored.

*Historical transaction data*. Historical transaction data here only refers to the records of consumers' purchasing behaviors on Tmall platform during a certain period of time. It includes detailed information such as transaction amount, transaction number, transaction path, and transaction time, which can further analyze consumers' purchasing patterns, purchasing habits, and the commonness between buyers.

*Consumer browsing logs*. When consumers visit different pages, different products, and different operations on Tmall platform, the server will record each request, which is called consumer browsing log. These logs contain a lot of information, including the IP address of the visitor, the page visited, the frequency of the visit, and the time of the visit.

*Product data*. Product data mainly refers to the attributes and characteristics of products including product specifications, price, and efficacy. Consumer purchasing power can be judged directly, and consumer preference can be judged indirectly based on product data.

## 3. Overview of Machine Learning Prediction Algorithms

Machine learning algorithms refer to learning certain rules or features by analyzing a large number of sample data so as to summarize, identify, and predict unknown results or unobservable data [21]. Its idea originated from modern statistical learning theory. However, the traditional statistical learning theory mainly provides important basis for limited sample learning, and its effect is not very ideal when the number of samples for analysis is large. Therefore, this paper selects the current popular machine learning algorithms for solving classification problems, mainly including logistic regression, random forest, support vector machine, and GBDT. Logistic regression model is widely used in classification learning due to its advantages of simple implementation, fast speed, and easy updating. Random forest model mainly reduces the risk of overfitting by means of average decision tree and makes the model relatively stable, so it is popular in machine learning. Support vector machine has become one of the most commonly used and most effective classifiers because of its excellent generalization ability. GBDT model is famous for its high prediction accuracy and flexible processing of various types of data. Each algorithm is not better than the others in every scenario, so try multiple algorithms for each application and evaluate the results.

### 3.1. Logistic Regression

Logistic regression model is actually based on linear regression, using Sigmoid function, it uses regression thinking to solve the problem of classification, and has been more widely used in the field of machine learning. Because of its simple principle and efficient classification, it is widely used in practice. Its idea comes from linear regression in statistics, and the difference is that its dependent variable is discrete [22]. The basic principle is that the output of dependent variable *y* in dichotomous problems can only be 0 or 1, instead of continuous values within a certain range. Therefore, in order to convert continuous output into a binary problem, continuous values can be converted into discrete binary values by nonlinear functions. *X*=〈*x*_1_, *x*_2_, ⋯, *x*_*n*_〉 is used to represent *n*-dimensional feature vectors, *n*-dimensional column vectors *θ*=〈*θ*_1_, *θ*_2_, ⋯, *θ*_*n*_〉 are used to represent corresponding weights or coefficients, and *b* is used to represent intercept. The linear relationship can be expressed as *F*(*θ*, *X*, *b*)=*θ*^*T*^*X*+*b*, where *F* ∈ *R*. When you are dealing with the simplest dichotomies, you need to map the value of *F* to (0, 1). A common Sigmoid function can be expressed as follows:

(1) $$\begin{array}{c} g\left(z\right)=\frac{1}{1+e^{-z}}, \end{array}$$

where *z* ∈ *R* and *g* is (0, 1).

The regularization of logistic regression is mainly to solve the problem of model overfitting, which is realized by modifying the cost function formula of algorithm, the expression of hypothesis function, and updating the iterative method so as to avoid overfitting of model and improve model generalization ability by punishing training samples of model [23]. During regularization operations, regularization coefficients can be set to *L*_1_ or *L*_2_, where the default value is *L*_2_.

(2) $$\begin{array}{c} J\left(\theta \right)=-\frac{1}{m}\sum_{i=1}^{m}\left[y^{\left(t\right)}\text{log}\left(h_{\theta }\left(x^{\left(t\right)}\right)\right)+\left(1-y^{\left(t\right)}\right)\text{log}\left(1-h_{\theta }\left(x^{\left(t\right)}\right)\right)\right]. \end{array}$$

The cost function of the logistic regression model is shown in formula (2), wherein the number of samples trained by the model is represented by *m*, the *y* value (true value) in the original training sample is still represented by *y*, *h*_*θ*_(*x*) represents the *y* value predicted by parameters *θ* and *x*, and the upper corner index (*t*) represents the *t* sample. The logistic regression minimization cost function *L*_2_ and logistic regression cost function *L*_1_ under regularization of binary classification are shown in the following formulas:

(3) $$\begin{array}{c} \underset{\omega ,c}{\text{min}}\frac{1}{2}\omega^{T}\omega +C\sum_{i=1}^{n}\log\left(\exp\left(-y_{i}\left(X_{i}^{T}\omega +c\right)\right)+1\right), \end{array}$$

(4) $$\begin{array}{c} \underset{\omega ,c}{\text{min}}{\left\|\omega \right\|}_{1}C\sum_{i=1}^{n}\log\left(\exp\left(-y_{i}\left(X_{i}^{T}\omega +c\right)\right)+1\right). \end{array}$$

### 3.2. Random Forest

Random forest belongs to the Bagging type. By combining multiple weak classifiers, the final result is obtained by voting or averaging, which can be used for demand prediction. The Bagging method is based on the following sampling rules: multiple samples are taken from the data set so that each sample may be taken twice or not at all, and the number of samples taken from each tree is the same and smaller than the number of samples in the original data. After sampling in this way, *n* decision trees can be constructed. Demand forecasting is a regression problem, and the final result can be obtained by means of taking the mean. Like other models, random forest can explain the effects of several independent variables *X*=〈*x*_1_, *x*_2_, ⋯, *x*_*k*_〉 on dependent variable *y*. The dependent variable *y* has *n* observation values, and the random forest will choose the method of bootstrap resampling to randomly select *n* observation values from the original data, among which some observation values have been selected many times, while others have not been selected. In addition, when constructing the classification tree, there are *k* independent variables related to it, and some variables are randomly selected from *k* independent variables to determine the node of the classification tree. Thus, the classification tree may be different each time it is built. In general, a random forest randomly generates hundreds to thousands of classification trees and then selects the tree with the highest degree of repetition as the final result [24].

**Figure 4:**
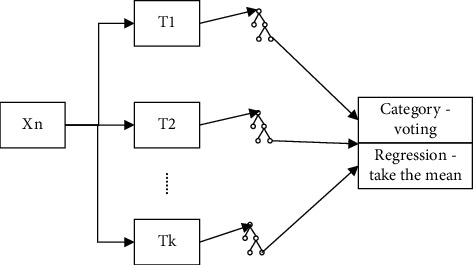
Random forest general structure.

Firstly, bootstrap method was applied to randomly select *K* new self-service sample sets from the original training data set, and *K* classification regression trees were constructed. The samples that were not selected each time constituted *K* out-of-bag data. Secondly, if there are *n* features, *m*_try_ feature (*m*_try_ ≤ *n*) is randomly selected at each node of each tree. By calculating the information contained in each feature, the feature with the most classification ability is selected for node splitting. Thirdly, maximize the growth of each tree without any clipping. Fourthly, the generated trees are formed into a random forest, which is used to classify the new data. The classification result is determined by the number of votes of tree classifier. The random forest general structure is shown in Figure 4.

### 3.3. Support Vector Machines

Support vector machine (SVM) is a kind of classifier based on the minimization of structural risk. By solving the quadratic programming problem, it seeks the optimal hyperplane that divides data into two types. Its theory originally comes from the processing of data classification problem. The principle of the support vector machine method can be simply described as to find an optimal classification hyperplane that meets the classification requirements so that the hyperplane can ensure the classification accuracy and maximize the blank area on both sides of the hyperplane so that the support vector machine can realize the optimal classification of linear frackable data [25]. Among them, the optimal classification plane should meet the following conditions:

(5) $$\begin{array}{c} \underset{\omega ,b}{\text{max}}\frac{2}{\left\|w\right\|}, \end{array}$$

(6) $$\begin{array}{c} s.t\;y_{i}\left[\left(w\cdot x_{i}\right)+b\right]-1\geq 0,\;i=1,2,\cdots ,n. \end{array}$$

**Figure 5:**
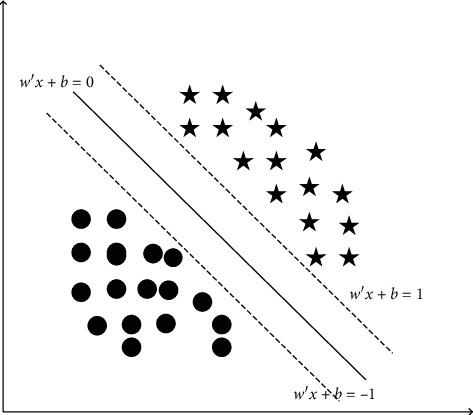
Schematic diagram of SVM.

In formula (5), 2/‖*w*‖ represents the size of the classification interval of the classifier. In order to make the classification plane robust in the classification process, it is usually necessary to find the maximum classification interval to achieve the optimal hyperplane [26]. In addition, in the constraint conditions, *y*_*i*_[(*w* · *x*_*i*_)+*b*] represents the distance between the sample point and the classification plane, and formula (6) indicates that the distance should be greater than 1. The basic principle of support vector machines can be understood more intuitively. The distance between the two dotted lines in the figure is the classification interval, the solid line with bold in the middle of the dotted line is the classification plane, and the black solid ball and black pentagram, respectively, represent the two types of samples to be classified. The schematic diagram of SVM is shown in Figure 5.

Kernel function is the core of the support vector machine. By introducing kernel function, we can realize nonlinear algorithm in a high-dimensional space. The kernel function of the support vector machine is the inner product of a high dimensional space, which plays an important role in the support vector machine [27]. Different kernel functions will generate different SVM algorithms. There are three kinds of kernel functions that are widely used: polynomial kernel function, radial basis kernel function, and neural network kernel function. The specific formula is as follows:

(7) $$\begin{array}{c} K\left(x_{i},y_{i}\right)={\left(x_{i}\cdot y_{j}+1\right)}^{q}, \end{array}$$

(8) $$\begin{array}{c} K\left(x_{i},y_{i}\right)=\text{exp}\left[-\frac{{\left|x_{i}-y_{j}\right|}^{2}}{\sigma^{2}}\right], \end{array}$$

(9) $$\begin{array}{c} K\left(x_{i},y_{i}\right)=\text{tan}h\left[c_{1}\left(x_{i}\cdot y_{j}\right)+c_{2}\right]. \end{array}$$

The principle of using structural risk in standard SVM is to select the error *ξ*_1_ (the relaxation variable allowing misclassification) as the loss function in the optimization objective. For classical support vector machines, the optimization problem is as follows:

(10) $$\begin{array}{c} \text{min}J\left(w,\xi \right)=\frac{1}{2}w\cdot w+c\sum_{i=1}^{l}\xi_{i}, \end{array}$$

(11) $$\begin{array}{c} s.t\;y_{i}\left[\left(\Psi \left(x_{i}\right)\cdot w+b\right)\right]\geq 1-\xi_{i},\;\xi_{i}\geq 0,\;i=1,2,\cdots ,l. \end{array}$$

Lagrange method was used to solve the above optimization problems, and the standard SVM optimization problem was transformed into the following quadratic programming:

(12) $$\begin{array}{c} \text{max}W\left(a\right)=-\frac{1}{2}\sum_{i,j=1}^{l}a_{i}y_{i}K\left(x_{i},x_{j}\right)a_{j}+\sum_{i=1}^{l}a_{i}, \end{array}$$

(13) $$\begin{array}{c} s.t\sum_{i=1}^{l}a_{i}y_{i}=0,\;0\leq a_{i}\leq c,\;i=1,2,\cdots ,l. \end{array}$$

### 3.4. GBDT

The gradient lifting decision tree is an iterative decision tree algorithm consisting of multiple decision trees, usually in the hundreds, each of which is small in size. Database marketing does not know enough about the potential market, development prospects, and the use of marketing methods. This makes the enterprise in the discussion and exploration stage for a long time. During this period, the direct value of database marketing only produced it, while its potential value was ignored [28]. The final predicted value of the test sample is the sum of the predicted values of the previous decision trees. In model prediction, an initial value will be given to an input sample instance, and then, each decision tree will be traversed. Each tree will adjust and modify the predicted value. The final result is to accumulate the results of each decision tree to get the final prediction result. The learning algorithm of GBDT includes regression algorithm and classification algorithm, which includes binary classification and multiclassification. Since this paper only deals with the problem of binary classification, only binary classification algorithms are introduced here. The loss function of dichotomies is defined as

(14) $$\begin{array}{c} L\left(y,f\left(x\right)\right)=\text{log}\left(1+\exp\left(-y\cdot f\left(x\right)\right)\right), \end{array}$$

where *y*_*i*_ ∈ *y*={−1, +1} . Then, the negative gradient error is

(15) $$\begin{array}{c} r_{mi}={\left[\frac{\partial L\left(y_{i},f\left(x_{i}\right)\right)}{\partial f\left(x_{i}\right)}\right]}_{f\left(x\right)=f_{m-1}\left(x\right)}\\ =\frac{y_{i}}{1+\exp\left(y_{i}\cdot f\left(x_{i}\right)\right)},\;i=1,2,\cdots ,N. \end{array}$$

For the generated decision tree, the best residual fitting value of each leaf node is

(16) $$\begin{array}{c} c_{mj}=\underset{c}{\text{argmin}}\sum_{x_{i}R_{mj}}\log\left(1+\exp\left(-y_{i}\left(f_{m-1}\left(x_{i}\right)+c\right)\right)\right). \end{array}$$

Regularization is mainly used to solve the overfitting phenomenon of models. For GBDT algorithm, its regularization processing methods can be summarized as the following three: one is the regularization setting by setting step parameters, which is similar to AdaBoost [29]. For the previous iterations of the weak learner, there are

(17) $$\begin{array}{c} f_{k}\left(x\right)=f_{k-1}\left(x\right)+vh_{k}\left(x\right), \end{array}$$

where *v* ranges from 0 ≤ *v* ≤ 1. When *v* is smaller, it means more iterations. Conversely, there are fewer iterations. Therefore, in the process of model construction, the step size and the maximum number of iterations are usually adjusted together to achieve a better fitting effect. Second, regularization is set by changing the subsample ratio. In general, the subsampling ratio ranges from 0 to 1. When the value is 1, it means full sampling, that is, all samples are used. When the value is less than 1, the corresponding samples are selected according to the proportion to fit the model to avoid overfitting. The third is regularization pruning with weak learner.

## 4. Analysis and Discussion of Calculation Results

**Figure 6:**
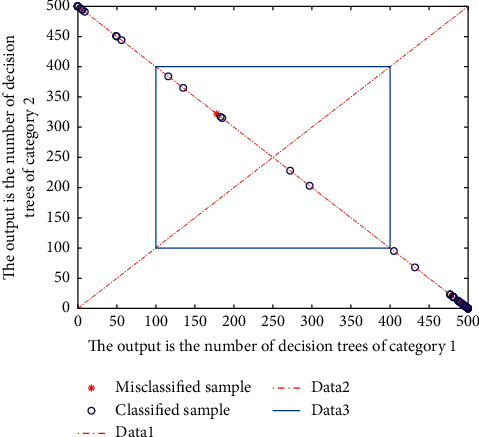
Random forest prediction result diagram.

The selection of feature variables is the key point of prediction model construction because the combination effect of feature variables directly affects the performance of the classification algorithm. At the same time, the selection of characteristic variables is also difficult [30]. First, because the number of feature variables is difficult to determine, if the number of feature variables is too small, the classification characteristics are not obvious, and the classification effect will be reduced. If the number of features is too much, it is easy to overfit and train the model for too long. Second, because of the complexity of features, different combinations of feature variables represent different classification information, but it requires constant attempts to combine feature sets with relatively good effects, which is time-consuming and costly. Thirdly, feature construction requires people with a strong sense of data and a high sensitivity to the industry. In the specific experimental process, because there is no specific theory to support, the characteristics of buildings are based on the understanding of store products, observation of consumer behavior, dynamic competition, cross category analysis, and through repeated practice and experience. Another simple method is to select all commonly used features and then screen them one by one by controlling variables. Based on the selection methods of two feature variables, this section will construct a feature system from the perspectives of consumer characteristics, store characteristics, and consumer-store interaction characteristics. The random forest prediction result diagram is shown in Figure 6.

**Figure 7:**
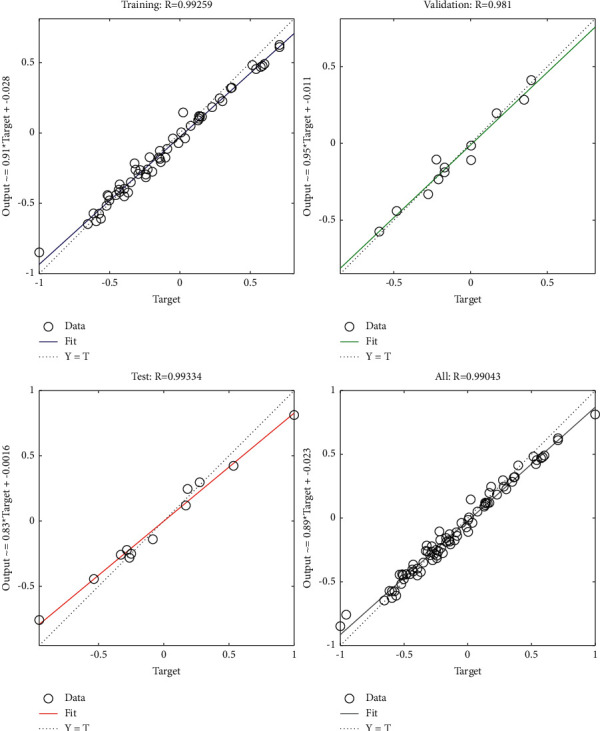
Test sample prediction renderings.

The test sample prediction renderings are shown in Figure 7. As we can see, GBDT algorithm has the best overall effect compared with other machine learning algorithms, among which GBDT model has the best performance. More than 50% of purchasing users (positive samples) are in the prediction score range of 80–99, and more than 75% of interactive nonpurchasing users (negative samples) are in the prediction score range of 0–9, accounting for more than 50%, indicating that positive and negative samples have a good prediction effect. At the same time, it is verified that the GBDT3 model selected by comparing the model effect is indeed excellent. The predicted sample is very close to the negative sample, and more than 64% of interactive nonpurchasing users (the predicted sample) are in the prediction score range of 0–9, which is close to the proportion of negative samples. At the same time, considering the similarity of the curve between the negative sample and the predicted sample, it can be judged that the model has a strong generalization ability and the prediction effect of the predicted sample is good. Therefore, in the actual business operation, the threshold value of the prediction sample is set within the prediction period of 80–99 as our prediction population, which is put into the application through the platform.

**Figure 8:**
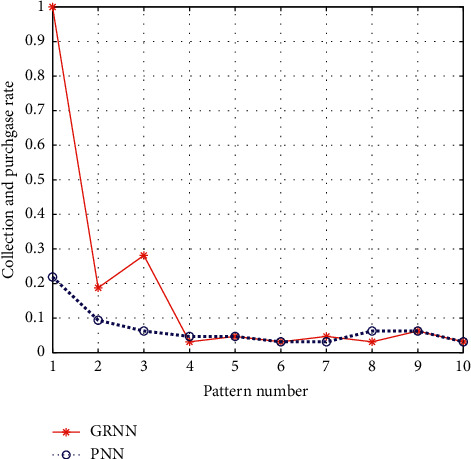
Model predicts the effect of population in different time periods.

This paper lists only 6 indicators according to the analysis needs, among which “display” refers to exposure times; Consumption refers to the store cost consumed by consumers through clicking advertisements; click rate = number of clicks/presentation; collection rate = (number of collected treasures + number of collected stores + number of added shopping carts)/visitors; turnover rate = volume of orders/visitors; ROI = amount of completed orders/consumption. For e-commerce, the most concerned indicator is ROI. When ROI = 1, it means that the amount of transaction is equal to the cost consumed by the store. When ROI is larger, it means that the amount of transaction is larger and the marketing effect is better. As can be seen from Figure 4, the ROI of the marketing method of crowd selection by model prediction is greater than 1 in all stages, with good performance, especially reaching the best in the outbreak period. The performance in the warm-up period is next, but the ROI can reach 3.5 under the condition of high consumption, and the effect is also good. In addition, although the ROI of the presale period is slightly lower than that of the other two stages, it brings a higher rate of collection and purchase, which plays an immeasurable role in the later transaction transformation. The model predicts the effect of population in different time periods which is shown in Figure 8.

**Figure 9:**
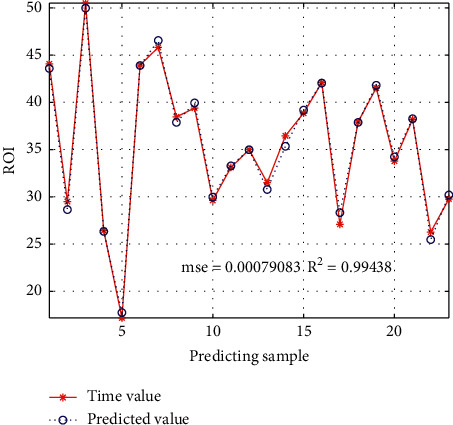
Different marketing methods of different effect comparison.

Next, the effect of model prediction is analyzed by comparing various marketing methods. Although the rate of collection and purchase of other groups (3) is good, their ROI is the lowest, indicating that certain traffic can be obtained through this marketing method, but the cost is high. The other group has the highest click-through rate of 9.29%, but the ROI is only 1.87. They can continue to optimize their placement by adding tags. Cross-category marketing consumption is the highest, mainly because there are too many choices of other categories with high correlation with mouthwash in the Lister flagship store. In this case, the ROI can be kept at 2.93, which indicates to some extent that cross-category marketing can still be used, but its operation process is too complicated. The collection and purchase rate of similar people is the highest, but the turnover rate is general. The turnover conversion rate and ROI of associate group are the best among all marketing methods, mainly because associate marketing is oriented to the old customers, and they have certain cognition, understanding, and loyalty to the product, so it is natural to rank first. The ROI predicted by the model was 4.51, second only to the combined population. The reasons are as follows: first, the group operation object mentioned above is the old customer; second, a careful observation of the combined population and the consumption predicted by the model shows that the consumption predicted by the model is much larger than that of the combined population, and the ROI predicted by the model is slightly smaller due to the formula for calculating ROI. The different marketing methods of different effect comparison are shown in Figure 9.

**Figure 10:**
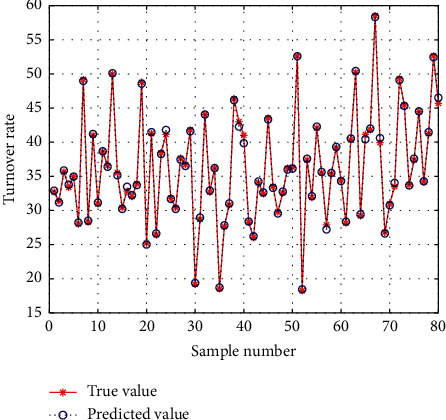
Effect comparison of different promotional activities.

Finally, the effect of model prediction is analyzed from the perspective of activity as a whole. The effect comparison of different promotional activities is shown in Figure 10. As shown in the figure, this article selects some electricity compared with 618 sales girl day activity; activity differs twice, one is due to the time of female group activity being relatively short, on the allocation of time compared to 618 fewer impoundment and preheating period, as a result of the model to predict marketing began reference to booking period, so there is little impact on the overall contrast. The second is that the 618 event adopts the marketing method of Queen's Day while adding the marketing method of model prediction. Locally, 618's presale period and outbreak period cost much more than The Queen's Festival, and in this case, 618's ROI still exceeded The Queen's Festival effect. In general, the collection and purchase rate, turnover conversion rate, and ROI of 618 promotion activities are also higher than the corresponding indicators of The Queen's Day.

## 5. Conclusion

Database marketing with big data revolution as the background, marketing theory as the basis, Internet as the media, and statistical methods as the means has become one of the development paths of major enterprises and electric stores. At home and abroad, database marketing continues to flourish, and database marketing based on machine learning algorithm is an important part. Therefore, this paper studies database marketing of an e-commerce company based on machine learning algorithm, and the main work and conclusions are summarized as follows:

1. Have an in-depth understanding of database marketing methods and choose machine learning algorithms to lay a foundation for subsequent model construction. The cognition of database marketing is mainly summarized from three levels of connotation, advantages, and basic procedures, while the machine learning algorithm is mainly for logical regression, random forest, support vector machine, and GBDT models, respectively, from the basic principle and algorithm introduction.
2. Analyze the current situation of an e-commerce company. After a brief introduction of the brand value and industry positioning of an e-commerce, the current situation of the store from the distribution of marketing objects, marketing channels, and marketing methods of three levels of the status of the description summed up the existing problems and put forward the database marketing ideas to solve the problems in the future.
3. This paper focuses on the construction of a multidimensional index system affecting the sales of e-commerce products and combines text mining with integrated learning methods to solve the sales forecasting problem of e-commerce products with small sample data. At the same time, a transfer learning model is constructed for the prediction of new product sales in the case of small samples and missing index data. This study provides an idea for e-commerce product sales forecast and a reference for e-commerce enterprises' demand decision-making.

This paper studies the database marketing of an ecommerce based on machine learning algorithm. In the process of research, it is supported by real database data, models, building platforms, and relevant statistical theories, but there is still no detailed query on some details. It is hoped that in the future, it can conduct in-depth research from the following aspects in combination with the actual situation of stores and the development of big data technology era:

1. Need to in-depth understanding of e-commerce database marketing status. As part of the data acquisition is difficult, and space limitations, this paper is mainly based on object database marketing, database marketing, and database marketing, three aspects to analyze the status quo, and the future can be further in other ways, only in understanding their own at the same time, to dig more potential commercial value, enrich the database marketing strategy, and at the same time to provide reference for other industries [31].
2. It is necessary to improve feature construction from multiple perspectives based on e-commerce practical experience. Feature selection itself is a complex problem. First, the complexity lies in the uncertain number of characteristic variables. When selecting too few characteristic variables, it is likely to cause data overlap and it is difficult to identify positive and negative samples. When there are too many characteristic variables and the data are too sparse, the same sample cannot be divided into the same category, resulting in inaccurate classifier. The 38 characteristic variables selected in this paper can be considered to be controlled in an appropriate range through experimental results. Second, complexity lies in the uncertainty of characteristic variables. The construction of characteristic variables requires comprehensive consideration of a variety of factors. Therefore, future research should focus on the specific selection of characteristic variables.
3. Need to expand machine learning prediction algorithms. As the e-commerce data factory has not been established for a long time, its operation interface is not very complete, and there are few models to choose from, and the operation of model fusion cannot be carried out. Therefore, in the future development and development process, users' purchasing behavior can be analyzed from multiple perspectives, and other models or model integration can be tried in terms of models.

## Data Availability

The data used to support the findings of this study are available from the author upon request.
